# Supplementary material for: Dynalign II: common secondary structure prediction for RNA homologs with domain insertions
Source: Nucleic Acids Res. 2014 Nov 21;42(22):13939–48. doi: 10.1093/nar/gku1172 (PMC4267632; doi:10.1093/nar/gku1172)
Supplement: SUPPLEMENTARY DATA [file supp_gku1172_nar-02021-z-2014-File012.zip › manual/RNA_class/html/class_r_n_a-members.html]

RNAstructure Classes: Member List

- Main Page
- Classes
- Files

- Class List
- Class Hierarchy
- Class Members

# RNA Member List

This is the complete list of members for RNA, including all inherited members.

|  |  |  |
| --- | --- | --- |
| AddComment(const char comment[], const int structurenumber=1) | RNA |  |
| BreakPseudoknot(const bool minimum\_energy=true, const int structurenumber=0) | RNA |  |
| CalculateFreeEnergy(const int structurenumber=1, const bool UseSimpleMBLoopRules=false) | RNA |  |
| ContainsPseudoknot(const int structurenumber) | RNA |  |
| CopyThermodynamic(Thermodynamics \*thermo) | Thermodynamics |  |
| ct | RNA | `[private]` |
| data | Thermodynamics | `[protected]` |
| DetermineDrawingCoordinates(const int height, const int width, const int structurenumber=1) | RNA |  |
| drawallocated | RNA | `[private]` |
| energyallocated | RNA | `[private]` |
| energyread | Thermodynamics | `[protected]` |
| enthalpy | Thermodynamics | `[protected]` |
| ErrorCode | RNA | `[protected]` |
| ev | RNA | `[private]` |
| ew | RNA | `[private]` |
| ew2 | RNA | `[private]` |
| ew3 | RNA | `[private]` |
| ew5 | RNA | `[private]` |
| ewmb | RNA | `[private]` |
| ewmb2 | RNA | `[private]` |
| fce | RNA | `[private]` |
| FileReader(const char filename[], const int type) | RNA | `[protected]` |
| FoldSingleStrand(const float percent, const int maximumstructures, const int window, const char savefile[]="", const int maxinternalloopsize=30, bool mfeonly=false) | RNA |  |
| ForceDoubleStranded(const int i) | RNA |  |
| ForceFMNCleavage(const int i) | RNA |  |
| ForceMaximumPairingDistance(const int distance) | RNA |  |
| ForceModification(const int i) | RNA |  |
| ForcePair(const int i, const int j) | RNA |  |
| ForceProhibitPair(const int i, const int j) | RNA |  |
| ForceSingleStranded(const int i) | RNA |  |
| GenerateAllSuboptimalStructures(const float percent, const double deltaG) | RNA |  |
| GetBackboneType() | RNA |  |
| GetCommentString(const int structurenumber=1) | RNA |  |
| GetDat(char \*loop, char \*stackf, char \*tstackh, char \*tstacki, char \*tloop, char \*miscloop, char \*danglef, char \*int22, char \*int21, char \*coax, char \*tstackcoax, char \*coaxstack, char \*tstack, char \*tstackm, char \*triloop, char \*int11, char \*hexaloop, char \*tstacki23, char \*tstacki1n, char \*datapath, bool isRNA, bool isEnthalpy=false) | Thermodynamics | `[protected]` |
| GetDatatable() | Thermodynamics |  |
| GetEnergyRead() | Thermodynamics |  |
| GetEnsembleEnergy() | RNA |  |
| GetEnthalpyTable() | Thermodynamics |  |
| GetErrorCode() | RNA |  |
| GetErrorMessage(const int error) | RNA |  |
| GetErrorMessageString(const int error) | RNA |  |
| GetForcedDoubleStranded(const int constraintnumber) | RNA |  |
| GetForcedFMNCleavage(const int constraintnumber) | RNA |  |
| GetForcedModification(const int constraintnumber) | RNA |  |
| GetForcedPair(const int constraintnumber, const bool fiveprime) | RNA |  |
| GetForcedProhibitedPair(const int constraintnumber, const bool fiveprime) | RNA |  |
| GetForcedSingleStranded(const int constraintnumber) | RNA |  |
| GetFreeEnergy(const int structurenumber) | RNA |  |
| GetLabelXCoordinate(const int i) | RNA |  |
| GetLabelYCoordinate(const int i) | RNA |  |
| GetMaximumPairingDistance() | RNA |  |
| GetNucleotide(const int i) | RNA |  |
| GetNucleotideXCoordinate(const int i) | RNA |  |
| GetNucleotideYCoordinate(const int i) | RNA |  |
| GetNumberOfForcedDoubleStranded() | RNA |  |
| GetNumberOfForcedFMNCleavages() | RNA |  |
| GetNumberOfForcedModifications() | RNA |  |
| GetNumberOfForcedPairs() | RNA |  |
| GetNumberOfForcedProhibitedPairs() | RNA |  |
| GetNumberOfForcedSingleStranded() | RNA |  |
| GetPair(const int i, const int structurenumber=1) | RNA |  |
| GetPairEnergy(const int i, const int j) | RNA |  |
| GetPairProbability(const int i, const int j) | RNA |  |
| GetProgress() | RNA |  |
| GetSequenceLength() | RNA |  |
| GetStructure() | RNA |  |
| GetStructureNumber() | RNA |  |
| GetTemperature() | Thermodynamics |  |
| isrna | Thermodynamics |  |
| lfce | RNA | `[private]` |
| MaximizeExpectedAccuracy(const double maxPercent, const int maxStructures, const int window, const double gamma=1.0) | RNA |  |
| mod | RNA | `[private]` |
| PartitionFunction(const char savefile[]="", double temperature=-10.0) | RNA |  |
| partitionfunctionallocated | RNA | `[private]` |
| pfdata | RNA | `[private]` |
| PredictProbablePairs(const float probability=0) | RNA |  |
| ProbKnot(int iterations=1, int MinHelixLength=1) | RNA |  |
| progress | RNA | `[protected]` |
| Q | RNA | `[private]` |
| ReadConstraints(const char filename[]) | RNA |  |
| ReadDSO(const char filename[]) | RNA |  |
| ReadExperimentalPairBonus(const char filename[], double const experimentalOffset, double const experimentalScaling) | RNA |  |
| ReadSHAPE(const char filename[], const double parameter1, const double parameter2, std::string modifier="SHAPE", const bool IsPseudoEnergy=true) | RNA |  |
| ReadSHAPE(const char filename[], const double parameter1, const double parameter2, const double ssm, const double ssb, std::string modifier="SHAPE") | RNA |  |
| ReadSSO(const char filename[]) | RNA |  |
| ReadThermodynamic(const char \*pathname=NULL) | Thermodynamics |  |
| ReFoldSingleStrand(const float percent, const int maximumstructures, const int window) | RNA |  |
| RemoveBasePair(const int i, const int structurenumber=1) | RNA |  |
| RemoveConstraints() | RNA |  |
| RemovePairs(const int structurenumber=1) | RNA |  |
| RNA(const char sequence[], const bool IsRNA=true) | RNA |  |
| RNA(const char filename[], const int type, const bool IsRNA=true) | RNA |  |
| RNA(const bool IsRNA=true) | RNA |  |
| SetExtrinsic(int i, int j, double k) | RNA |  |
| SetModifier(std::string modifier) | RNA |  |
| SetProgress(TProgressDialog &Progress) | RNA |  |
| SetTemperature(double temperature) | Thermodynamics |  |
| SpecifyPair(const int i, const int j, const int structurenumber=1) | RNA |  |
| Stochastic(const int structures, const int seed=1) | RNA |  |
| StopProgress() | RNA |  |
| structurecoordinates | RNA | `[private]` |
| temp | Thermodynamics | `[protected]` |
| Thermodynamics(const bool ISRNA=true) | Thermodynamics |  |
| v | RNA | `[private]` |
| vmin | RNA | `[private]` |
| w | RNA | `[private]` |
| w3 | RNA | `[private]` |
| w5 | RNA | `[private]` |
| wca | RNA | `[private]` |
| wcoax | RNA | `[private]` |
| wl | RNA | `[private]` |
| wmb | RNA | `[private]` |
| wmbl | RNA | `[private]` |
| WriteConstraints(const char filename[]) | RNA |  |
| WriteCt(const char filename[], bool append=false) | RNA |  |
| WriteDotBracket(const char filename[]) | RNA |  |
| WriteThermodynamicDetails(const char filename[], const bool UseSimpleMBLoopRules=false) | RNA |  |
| ~RNA() | RNA |  |
| ~Thermodynamics() | Thermodynamics |  |

---

Generated on Tue Jul 30 10:52:29 2013 for RNAstructure Classes by 
 1.5.7.1
